# Influence of dietary mealworm (*Tenebrio molitor*) on nutrient digestibility, performance, and blood constituents in growing lambs

**DOI:** 10.1007/s11250-026-05157-9

**Published:** 2026-06-22

**Authors:** Hanan A. M. Hassanien, Mohammed A. Abu ElKassim, Hamdan M. Tawfik, Waleed K. Abouamra, Ekramy H. Hassan, Mohamed A. Radwand, Ofelia Márquez-Molina, Abdelfattah Z. M. Salem

**Affiliations:** 1 Animal Production Research Institute (APRI), Agricultural Research Center (ARC), Giza, 12619 Egypt; 2 Department of Animal Production, Faculty of Agriculture, Al-Azhar University, Assiut, 71524 Egypt; 3 Department of Animal Production, Faculty of Agriculture, Cairo University, Giza, Egypt; 4 Centro Universitario UAEM Amecameca, Universidad Autónoma del Estado de México, Amecameca, México; 5 Dipartimento di Scienze del Suolo, della Pianta e degli Alimenti (Di.S.S.P.A.), Università degli Studi di Bari, Via Giovanni Amendola, 165/a, Bari, 70126 BA Italy

**Keywords:** Sustainability, Mealworm, Larvae, growth, Digestibility, Profitability, Lambs

## Abstract

The present study aimed to evaluate the effect of dietary inclusion of mealworms (*Tenebrio molitor*) in the form of larvae and whole insects on growth performance, rumen fermentation parameters, selected health indicators, and economic efficiency in growing Ossimi lambs. Eighteen Ossimi lambs, aged 4 to 5 months with an initial weight of 23.7 ± 2 kg, were arbitrarily allocated into three identical groups. The control group was fed a basal ration consisting of a concentrate feed combination at 2% according to body weight and fed wheat straw freely, without any additives; the larvae mealworm (LMM) group was provided with a basal diet supplemented with (1g/kg) of LMM powder; and the insect mealworm (IMM) group was administered a basal diet enhanced with (1g/kg) of IMM powder. At the beginning of the trial, animals were weighed in the morning before feeding and every two weeks during the experimental period (120 days). The findings indicated that the LMM and IMM groups exhibited increased final BW, total gain, and daily gain increase relative to the control group, but the differences were not significant. The feed efficiency based on dry matter (DM) and total digestible nutrients was comparable across the three groups. However, digestible crude protein was considerably elevated (*P* = 0.024) in group IMM relative to the control and other groups. The LMM and IMM groups had considerably greater amounts of DM, organic matter, and carbohydrates than the control group. In the control group, blood albumin and glucose levels were higher, whereas blood globulin levels were lower compared to the LMM and IMM groups (*P* < 0.05). The economic return and return on investment were numerically higher in the LMM and IMM groups in comparison with the control group. In conclusion, the inclusion of mealworms (either larvae or complete insects) in ruminant diets may improve animal production and profitability without negatively impacting animal health.

## Introduction

The world’s food production systems are facing considerable strain, largely because of the swift expansion of the human population. Projections suggest that the global population will reach 9 billion by 2050 (Opio et al. 2013). As a result, this population increase is expected to lead to a 58% rise in milk consumption and a 70% increase in the demand for beef, compared to the levels seen in 2010. One of the major challenges facing global animal husbandry operations is the cost of feed, which accounts for over 70% of the overall production budget, with more than 15% allocated to fulfilling dietary protein requirements (Khan et al. 2016; van Huis 2013). Soybean meal, notwithstanding its elevated crude protein levels and well-balanced amino acid composition, continues to be a favored protein source for ruminants (Jolazadeh et al. 2015). Nevertheless, the sustainable application of soybeans faces limitations stemming from fluctuating market prices, the inherent instability of global supply chains, and the significant environmental impacts linked to soybean cultivation (Halloran et al. 2018; FAO 2021). Consequently, there is a growing demand for protein sources that are both environmentally responsible and economically feasible. Within this framework, insect meal has emerged as a favored eco-friendly protein and fat source for livestock feed. Insect farming presents several benefits within a circular economy, as insects possess the capacity to convert organic waste into nutrients that are advantageous for both human and animal consumption (Tomberlin et al. 2015; United Nations Global Compact and KPMG, 2016). Research has consistently demonstrated that insects, including mealworms (*Tenebrio molitor*), are high in crude protein and frequently exhibit superior nutritional profiles compared to traditional feed components such as soybean meal (Klunder et al. 2012; Henchion et al. 2017; Kim et al. 2019). Additionally, the complete proteins found in insects are just as easily digested as those found in eggs, milk, or meat (Shockley and Dossey 2014). Consequently, the utilization of agricultural by-products for insect production mitigates waste and bolsters the sustainability of bio-recycling, a crucial element of environmentally sound agricultural methodologies.

Although the advantages of insect meals for monogastric animals are extensively documented, their integration into ruminant diets is still a relatively novel area of research. Presently, there is no substantiated evidence indicating that insects can serve as reservoirs for prions or facilitate their transmission. Nonetheless, historical apprehensions regarding bovine spongiform encephalopathy (BSE) have prompted the restriction or prohibition of insect-derived components in ruminant feeds across numerous nations (DiGiacomo and Leury 2019). Furthermore, insects demonstrate low ruminal digestibility unless subjected to pre-treatment or processing to enhance nutrient accessibility (Renna et al. 2022a). Limited research has assessed the viability of insect meal as an alternative protein source for beef steers (Fukuda et al. 2022), goats (Astuti et al. 2019), and under in vitro rumen fermentation conditions (Jayanegara et al. 2017a, b, 2020). Recent laboratory studies have examined the effects of adding mealworm meal to the diet, along with a natural tannin source like *Acacia farnesiana* L., on rumen fermentation dynamics, dry matter disappearance, and gas production. These findings also suggest potential enhancements in protein utilization in sheep (Gonzalez-Ronquillo et al. 2025). In line with these emerging studies, Parodi et al. (2022) have emphasized the significant role of insect-derived proteins in enhancing sustainable livestock production. Their framework advocates for the integration of insect meals, specifically those derived from mealworms, as environmentally responsible feed options, aligning with global goals for food security and reduced ecological impact. Furthermore, Parodi et al. (2022) highlight the broader ecological benefits of using insect-based proteins, such as lower land, water, and feed requirements. These findings are consistent with the potential environmental benefits of substituting conventional feed ingredients such as soybean meal with insect-derived proteins. Furthermore, Renna et al. (2023) emphasised the importance of additional research to achieve precise nutritional value of insect-derived products (whole fat, defatted meals, oils, and other by-products), and to determine optimal inclusion rates for various ruminant species. The incorporation of mealworm larvae into ruminant diets has demonstrated enhancements in growth performance, rumen metabolism, and overall health (Gasco et al. 2019; Renna et al. 2022b; DiGiacomo and Leury 2019). These findings suggest that insects may represent a financially viable and ecologically sustainable feeding approach. Toral et al. (2022) observed that *Tenebrio molitor* displayed the least ruminal nitrogen degradation and the most favorable intestinal digestibility among the four insect species examined. This supports its potential as a promising substitute for soybean meal in ruminant feed formulations. Overall, available evidence indicates that the inclusion of mealworm in ruminant diets is safe, and no adverse effects on health, rumen metabolism, or growth performance, thus contributing to decreased feed expenses and enhanced sustainability. The main goal of this study is to assess the effects of including mealworm (*Tenebrio molitor*) larvae and whole insects in the diets of Ossimi lambs during their growth. The study specifically seeks to determine the effects of this dietary inclusion on various aspects, including growth performance, rumen fermentation characteristics, health markers, and economic viability. It was hypothesized that supplementing lamb diets with Tenebrio molitor would enhance growth performance and nutrient digestibility without adverse effects on health or rumen fermentation. Both larvae and whole insects were evaluated to compare their distinct nutritional profiles—specifically the higher fat content in larvae versus the potentially higher chitin and protein levels in adults—to determine the most effective life stage for ruminant supplementation. Therefore, the present study investigated the effects of dietary supplementation with *Tenebrio molitor* larvae meal or whole insects on growth performance, feeding efficiency, and blood biochemical parameters of Ossimi lambs, in order to explore their potential as a sustainable protein supplement in ruminant nutrition.

## Materials and methods

### Animal ethical approval

The animals in this experiment were approved (No. ARC APRI 43 25) through the APRI Ethical Committee, Agricultural Research Center, Egypt. This research was performed at the Animal Production Department’s Research Farm at Al-Azhar University in Assiut, Egypt.

### Experimental animals and housing conditions

Eighteen male Ossimi lambs (23.7 ± 2 kg initial BW; 4–5 months old) were arbitrarily allocated into three treatment groups (*n* = 6 per group). The lambs were group-housed in a semi-open shed in pens (approximately 1.8 m^2^ per lamb) with concrete floors covered by wheat straw bedding, which was refreshed every two days. Each pen was equipped with wooden feed bunks of sufficient length to allow all lambs to feed simultaneously, and automatic stainless-steel waterers provided continuous access to fresh water. While the number of animals per group was determined by the facility’s capacity for controlled group-feeding, the study ensured high data reliability by using the group as the experimental unit for feed intake and efficiency, simulating commercial field conditions in Egypt.

### Diet formulation and feeding protocol

The experimental rations were formulated to meet the maintenance and growth requirements of growing lambs according to the National Research Council (NRC, 2007), targeting a daily weight gain of 150–200 g and specific dry matter and crude protein intake levels for this age group. The control group received a basal diet consisting of a concentrate feed mixture (CFM) at 2% of body weight (BW) and wheat straw *ad libitum*. The other two groups were fed the same basal diet supplemented with 1g/kg of either larvae mealworm powder (LMM) or whole insect mealworm powder (IMM). Feeding behavior was monitored through daily observation of feed bunk clean-out and competition levels during the two equal meal offerings at 08:00 and 15:00. The CFM amount was adjusted bi-weekly based on live BW changes recorded before morning feeding throughout the 120-day trial to maintain targeted nutrient density. The chemical composition of the rations is detailed in Table 1.

**Table 1:** Ingredients and chemical composition of CFM, LMM, IMM and diets (% as DM basis)^1^

Item	CFM^1^		Experimental diet		Tenebrio molitor	
		WS	LMMP	IMMP	LMM	IMM
Dry matter	88.88	95.50	97.10	87.10	89.02	88.77
Organic matter	88.88	86.10	91.13	91.43	88.94	88.92
Crude protein	14.05	3.10	43.38	29.28	14.49	14.34
Crude fiber	19.22	27.30	9.28	24.75	19.12	19.47
Ether extract	4.08	2.70	19.67	12.50	4.26	4.20
Nitrogen-free extract	51.53	53.00	18.8	24.09	51.20	51.13
Ash	11.12	13.88	8.87	8.57	11.06	11.08

^1^CFM: Concentrate feed mixture; WS: Wheat straw; LMMP: Mealworm larva powder; IMMP: Insect of mealworm powder; LMM: LMM: basal diet with additive (g mealworm larva /kg ration); IMM: basal diet with additive (g insect of mealworm/kg ration)

### Digestibility experiment

Three digestion experiments were applied after the growth trial, using nine mature rams weighing 48 ± 0.5 kg on average (three animals per treatment). This experiment aimed to determine the nutritional value of experimental diets by assessing key digestibility traits concerning digestible crude protein (DCP) and total digestible nutrients (TDN). Rams were housed in metabolic cages with unlimited access. The CFM was administered in amounts at 9:00 am and 2:00 pm, followed by roughages (Maynard et al. 1979). The experiment consisted of an initial week-long phase followed by an additional week for sample collection. Feed, refusals, and feces samples were collected in the morning and stored in a frozen state till analysis. Feces samples were oven-dried at 50–60 °C for 12 h. Dried feed, refusals, and feces samples that were collected for five days were mixed for chemical analysis (AOAC 2007). Following the digestibility test, a stomach tube was used to collect the rumen fluid samples from all animals. The samples were collected at two times: immediately before feeding (0 h) and post-feeding (4 h). Four layers of cheesecloth were subjected to filtration of the samples. Ruminal pH levels were determined by a HANNA pH meter (HI8424). Concentration of NH_3_-N and total volatile fatty acids (TVFA) in rumen liquor samples were conducted according to (AOAC 2007) and (Warner 1964), respectively.

### Blood parameters

Following (Palo et al. 2018), blood was drawn in the early morning before feeding from the jugular vein in heparinized Vacutainer^®^ tubes. Samples were exposed to centrifugation at 3000 x g for 20 min; the plasma fraction was immediately frozen at -20 °C pending analysis. The levels of total protein, urea, creatinine, cholesterol, triglyceride, glucose, albumin, and globulin were determined using colorimetric kits from Diamond Diagnostics, Egypt.

### Economic evaluation

The economic efficiency of the experimental rations was calculated based on the prevailing local market prices of feed ingredients and the live body weight of lambs at the time of the study. The total feed cost for each group was determined by multiplying the total feed intake by the cost of each ingredient. The following parameters and formulas were used to assess the economic impact of mealworm supplementation:

$$\eqalign{ & {\rm{Total\,Feed\,Cost}}\left( {{\rm{LE}}/{\rm{lamb}}} \right)\, = \,{\rm{sum}} \cr & \left( {{\rm{Feed\,in\,take\,of\,each\,ingredient}} \times {\rm{Price\,of\,ingredient}}} \right) \cr}$$

$$\eqalign{ & {\rm{Total}}\,{\rm{Revenue}}\left( {{\rm{LE}}/{\rm{lamb}}} \right){\rm{}} = {\rm{Total}}\,{\rm{weight\,gain}}\left( {{\rm{kg}}} \right) \cr & {\rm{}} \times {\rm{Price}}\,{\rm{of}}\,1{\rm{kg}}\,{\rm{of}}\,{\rm{live}}\,{\rm{body}}\,{\rm{weight}} \cr}$$

$$\eqalign{{\rm{Net\,Revenue}}\left( {{\rm{LE}}/{\rm{lamb}}} \right){\rm{}} & = {\rm{}}\left( {{\rm{Total\,Revenue}}} \right) \cr & {\rm{}} - {\rm{Total\,Feed\,Cost}} \cr}$$

$$\eqalign{{\rm{Economic}}\,{\rm{Efficiency}}\left( {{\rm{EE}}} \right)\, & = \,{\rm{Net}}\,{\rm{Revenue}} \cr & {\rm{}}/{\rm{Total}}\,{\rm{Feed}}\,{\rm{Cost}} \cr}$$

$$\eqalign{ & {\rm{Relative\,Economic\,Efficiency}}\left( {{\rm{REE}},{\rm{\% }}} \right)\, \cr & = \,{\rm{EE\,of\,the\,treatment\,group}} \cr & /{\rm{EE\,of\,the\,control\,group}} \times {\rm{}}100 \cr}$$

All prices were calculated in Egyptian Pounds (LE) according to the market conditions during the 120-day trial period.

### Statistical analysis

Data were analysed using the general linear model (GLM) procedure by SAS (2009). The percentage values were transformed before analysis. The significance of differences among the three groups was assessed by Duncan’s multiple at α = 0.05 (Duncan 1955). The statistical model is expressed as: Y_ij_ = µ + R_i_ + e_ij_. In this context, Y_ij_ represents the observation for _ij_, µ denotes the overall mean of Y_ij_, R_i_ signifies the impact of i (rations), and e_ij_ represents the random error.

## Results

### Feed chemical compositions

Table 1 presents the chemical composition of CFM, LMMP, IMMP, and diets containing LMM and IMM. LMMP and IMMP contain (43.38% and 29.28%) crude protein (CP), respectively. Crude fiber (CF) was higher in IMMP (24.75%) than in LMMP (9.28%), whereas ether extract (EE) was higher in LMMP (19.67%) than in IMMP (12.50%).

### Digestibility coefficients and nutritive value

The present findings (Table 2) demonstrate that the inclusion of LMMP and IMMP in lamb diets significantly enhanced the digestibility of DM, OM, EE, carbohydrates and TDN relative to the control group (*P* < 0.05).

**Table 2:** Effect of experimental lamb rations^1^ on nutrient digestibility and nutritive value

Item^2^	Control	LMM	IMM	SEM	*P* value
Digestibility Coefficients, %					
Dry matter	56.2^b^	63.0^a^	62.4^a^	1.18	0.012
Organic matter	61.2^b^	66.9^a^	66.6^a^	1.08	0.017
Crude protein	65.2	69.5	69.3	2.01	0.316
Crude fiber	60.4	63.4	61.5	2.16	0.634
Ether extract	56.0^b^	62.7^ab^	64.8^a^	1.84	0.034
Carbohydrate	60.9^b^	67.9^a^	68.0^a^	1.31	0.013
Nutritive value^2^					
DCP %	7.4	7.9	8.6	0.8	0.589
TDN%	56.1^b^	61.4^a^	61.1^a^	1.0	0.013

^1^ LMM: basal diet with additive (g mealworm larva /kg ration); IMM: basal diet with additive (g insect of mealworm /kg ration)

^2^ TDN: total digestible nutrients; DCP: digestible crude protein. Means in the same row with different superscripts (a, b) are significantly different (*P* < 0.05). SEM: Standard error of the mean

### Growth performance and feed utilization efficiency

The data presented in Table 3 demonstrate that lambs fed the LMM and IMM diets achieved higher numerical final body weight, total weight gain, and average daily gain (ADG) compared to the control group. However, these differences were not statistically significant (*P* > 0.05). Although the consumption of CFM, roughage, total dry matter (DM), and total digestible nutrients (TDN) were similar across all treatments. Although the IMM group exhibited numerically higher DCP values, the differences were not statistically significant.

**Table 3:** Growth performance and feed efficiency of lambs fed on different experimental rations^1^

Item^2^	Control	LMM	IMM	SEM	*P* value
Growth performance:					
Initial body weight, kg	23.6	23.7	23.8	0.9	0.990
Final body weight, kg	39.5	40.1	40.5	1.0	0.780
Total body weight gain, kg	15.8	16.4	16.7	0.5	0.411
Average daily gain, g	132.0	136.7	139.3	3.8	0.411
Feed intake as fed, kg					
CFM, kg	62.8	61.5	68.3		
Roughage, kg	30.5	30.7	33.1		
Total feed intake, kg	93.4	92.2	101.2		
DM intake, kg	84.1	83.0	91.1		
TDN intake, kg	47.2	50.9	55.7		
DCP intake, kg	6.2	6.5	7.8		
Feed conversion ratio:					
kg DM /1kg gain	5.3	5.1	5.5		
kg TDN/1kg gain	3.0	3.1	3.3		
kg DCP/1kg gain	0.39	0.40	0.47		

^1^LMM: basal diet with additive (g mealworm larva /kg ration); IMM: basal diet with additive (g insect of mealworm /kg ration)

^2^CFM: concentrate feed mixture; DM: dry matter; TDN: total digestible nutrients; DCP: digestible crude protein

Means in the same row with different superscripts (a, b) are significantly different (*P* < 0.05). SEM: Standard error of the mean

### Ruminal fermentation

**Fig. 1:**
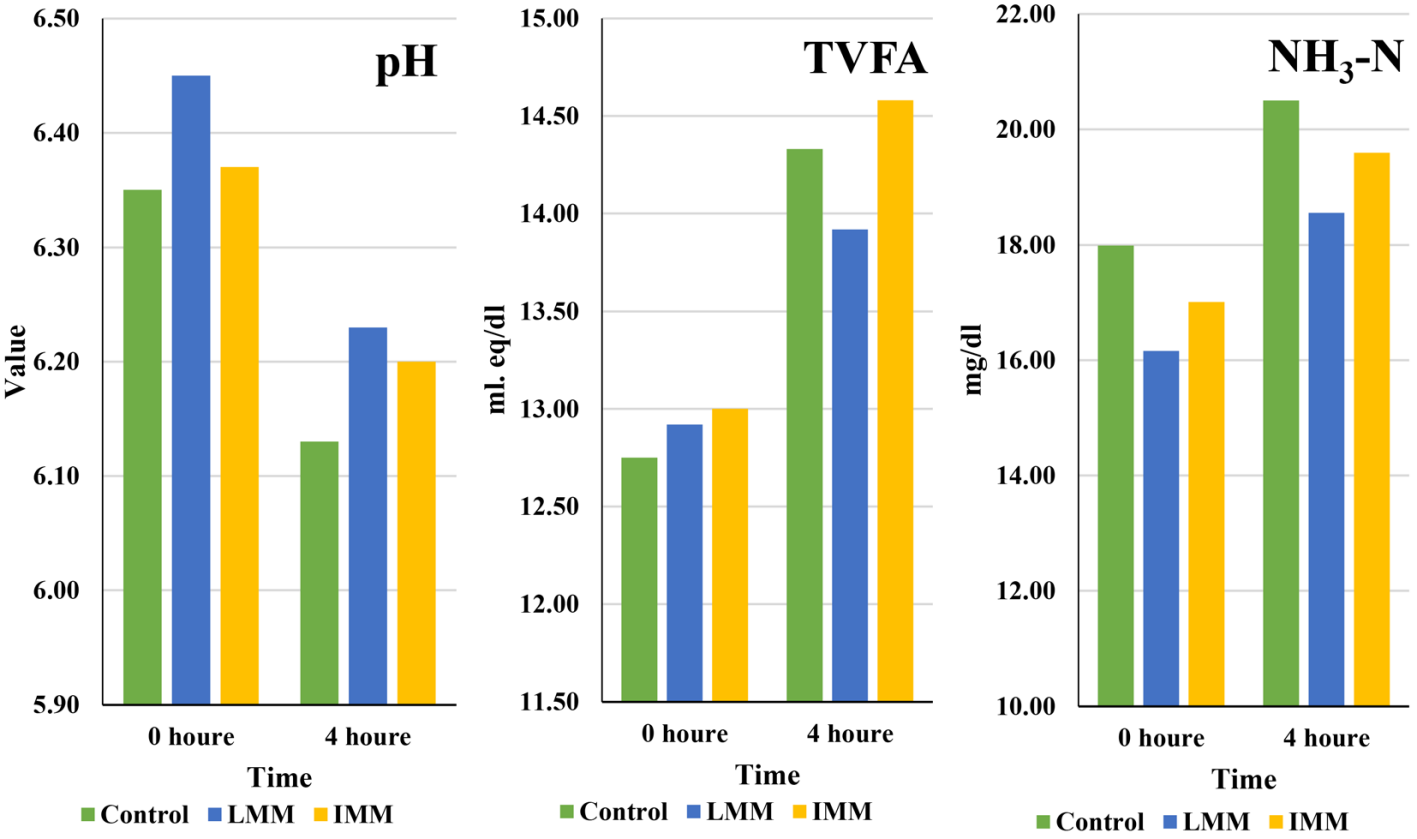
The influence of experimental rations on rumen parameters after 0 and 4 h of feeding. LMM: basal diet with additive (g mealworm larva /kg ration); IMM: basal diet with additive (g insect of mealworm /kg ration. TVFA: total volatile fatty acids

Figure 1 illustrates rumen parameters at zero- and four-hours post-feeding. Rumen pH levels decreased in all groups at 4 h post-feeding. The control group showed a declin from 6.35 to 6.13, the LMM group from 6.45 to 6.23, and the IMM group from 6.37 to 6.2. This suggests a minor drop in pH over time across all rations, which is consistent with the observed increase in total volatile fatty acids (TVFA). There was an increase in TVFA levels after 4 h in all groups. The control group showed an increase from 12.75 to 14.33, the LMM group from 12.92 to 13.92, and the IMM group from 13.0 to 14.58. This indicates a general rise in fermentation activity over time. The ammonia levels increased in the control and IMM groups but slightly decreased in the LMM group after 4 h. The control group had a rise from 17.99 mg/dl to 20.5 mg/dl, while LMM increased from 16.16 mg/dl to 18.55 mg/dl, and IMM rose from 17.01 mg/dl to 19.59 mg/dl. This highlights that LMM may have contributed to a slightly more efficient use or absorption of ammonia.

### Blood indicators

Table 4 shows the impact of LMM and IMM on biochemical blood parameters. No substantial variations were seen in most blood markers among the groups. The control group exhibited higher albumin levels compared to the LMM and IMM groups, with a statistically significant difference (*P* = 0.035). Both the LMM and IMM groups showed higher (*P* = 0.012) globulin levels than the control group. The albumin/globulin ratio was significantly (*P* = 0.033) lower in the LMM and IMM groups compared to the control. The IMM group showed significantly (*P* < 0.0001) reduced glucose levels compared to the control and LMM groups. No significant differences were observed in triglycerides, cholesterol, creatinine, or urea levels among the groups.

**Table 4:** Some biochemical concentrations in the blood plasma of lambs fed experimental rations^1^

Item	control	LMM	IMM	SEM	*P* value
Total protein, g/dl	6.22	6.71	7.11	0.29	0.133
Albumin, g/dl	3.80^a^	3.32^b^	3.57^ab^	0.12	0.035
Globulin, g/dl	2.42^b^	3.39^a^	3.54^a^	0.25	0.012
Albumin/ globulin ratio	1.66^a^	1.00^b^	1.05^b^	0.13	0.033
Glucose, mg/dL	98^a^	92^a^	76^b^	2.44	< 0.0001
Triglyceride, mg/dL	209	212	190	8.37	0.176
Cholesterol, mg/dL	216	217	199	21.65	0.800
Creatinine, g/dl	1.88	1.51	1.53	0.19	0.322
Urea, mg/dL	40.5	40.3	38.5	1.22	0.457

^1^LMM: basal diet with additive (g mealworm larva /kg ration); IMM: basal diet with additive (g insect of mealworm /kg ration)

Means in the same row with different superscripts (a, b) are significantly different (*P* < 0.05). SEM: Standard error of the mean

### Economic efficiency

Table 5 illustrates that there were no significant differences among the three groups; nevertheless, both the LMM and IMM groups exhibited numerically higher return on investment and economic profit relative to the control group. The return on investment was 29.3% for the control, 30.9% for LMM, and 32.3% for IMM, with no significant differences detected (*P* = 0.673). The LMM group demonstrated an economic efficiency of 105.6%, whereas the IMM group showed a slightly diminished efficiency of 104.5%, both surpassing the control group’s 100%.

**Table 5:** The economic efficiency of lambs fed on different experimental rations^1^

Item	Control	LMM	IMM	SEM	*P* value
Lamb purchasing cost, L.E/head	5292	5208	5064	209	0.743
Total feed cost, L.E./ head	972	981	996	41	0.917
Total costs, L.E./ head	6264	6189	6060	249	0.844
Feed cost/Kg gain, L.E.	59.2	59.3	58.6	2.6	0.981
Income, L.E./head ^A^	8092	8057	8001	199	0.948
Economical return, L.E./head ^B^	1827	1868	1940	82	0.628
Return on investment, % ^C^	29.3	30.9	32.3	2.4	0.673
Relative economic efficiency, % ^D^	100	105.6	104.5	-	-

^1^LMM: basal diet with additive (g mealworm larva /kg ration); IMM: basal diet with additive (g insect of mealworm /kg ration). ^A^ Income = lamb final body weight* price of one kg (200 LE). ^B^ economical return= Income – total cost. ^C^ Return on investment (%) =economic return (L.E)/total costs *100. ^D^ Relative economic efficiency (%) = Return on investment (treatment)/ Return on investment (control)*100.Where: prices as follows; concentrate feed mixture = 12,500 L.E.\ton, wheat straw = 4500 L.E.\ton and price of 1kg of purchasing lamb = 220 L.E

## Discussion

### Feed chemical compositions

The observed compositional differences between LMMP and IMMP (Table 1) highlight the effect of insect growing stage on nutritional profile and potential functionality in ruminant diets. The higher CP and EE contents in LMMP confirm that mealworm larvae signify a concentrated source of both protein and energy. These findings matched with previous reported ranges for *Tenebrio molitor* larvae (Ravzanaadii et al. 2012; Syahrulawal et al. 2023), supporting the consistency of their nutritional value across studies. But, while the raised protein content supports their consumption as a replacement to conventional protein sources, the real efficiency of protein usage in practical feeding systems permits further in-vivo validation.

In contrast, the higher CF value in IMMP probably reflects increased chitin deposition associated with exoskeletal maturation. The chitin content of dried larvae mealworms has been reported at 3.6–6.3% (Muñoz-Seijas et al. 2024; Sudwischer et al. 2025), whereas mealworm beetles contain around 22.7% (Muñoz-Seijas et al. 2024). Although chitin is often considered a limiting factor due to its reduced digestibility, initial evidence suggests it may exert modulatory effects on rumen microbial populations (Rossi et al. 2022; Carrasco and Drewery 2024). Therefore, the elevated fiber fraction in IMMP should not be interpreted only as a nutritional disadvantage but somewhat as a potential functional component influencing fermentation dynamics. The balance between reduced digestibility and possible prebiotic effects remains an area requiring further investigation.

The elevated EE content detected in LMMP also matches with literature representing that larvae own greater lipid retains, specifically unsaturated fatty acids like oleic and linoleic acids (Renna et al. 2022b). So, this may improve nutritional energy mass and support animal performance (growth or lactation), but excessive fat insertion could adversely affect rumen fermentation if not correctly balanced. Thus, optimal insertion rates remain vital.

### Digestibility coefficients and nutritive value

Table 2 shows substantial variations in nutrient digestibility and nutritional value between the control group and LMMP/IMMP supplemented groups. The enhanced digestibility in the LMM and IMM groups suggests that insect-based meals improve ruminant nutrient absorption, supporting prior *Tenebrio molitor* investigations in livestock feed. LMM and IMM exhibited considerably higher digestibility of DM, OM, and Car than the control group. The results support Renna et al. (2022b) and Shah et al. (2022) who use of mealworm larvae in ruminant diets to improve nutrient absorption. Our findings corroborate Abdel Hakeam et al. (2024) studies on Ossimi lambs’ diets with insect meal instead of soybean meal. Their findings showed that insect meal supplementation didn’t impede nutritional digestion and even improved it. Robles-Jimenez et al. (2025a) found that *Tenebrio molitor* larvae improved rumen digestion and nutritional absorption in ruminants. Ruminantes fed mealworm larvae had higher rumen fermentation, nutritional absorption, and lamb growth rates. As Syahrulawal et al. (2023) reported the mealworm protein contains essential amino acids such lysine, leucine, and isoleucine, which are vital for ruminant health. Further experiments should be conducted to assess the long-term influences of insect meal on rumen health and microbial profile. Digestibility of CF was numerically higher in the LMM and IMM groups. However, the IMMP had more fibre (24.75%) than the LMMP (9.28%), but the IMM group had slightly better CF digestibility than the LMM group and the control group. The high chitin content in IMMP may improve ruminal fermentation and microbial activity, which could contribute to the observed differences in CF digestibility among the groups. Chitin in insect exoskeletons has prebiotic, antibacterial, and immunomodulatory properties that promote the growth of beneficial rumen microbes (Zanferari et al. 2018; Gasco et al. 2019; Shah et al. 2022; Rossi et al. 2022; Syahrulawal et al. 2023). EE digestibility was greater in the IMM group (64.8%) compared with the control group (56.0%). Renna et al. (2022b) found that full-fat insect meals had improved lipid digestibility. They found that *Tenebrio molitor* larvae contain oleic acid (C18:1) and linoleic acid (C18:2), which are required for ruminant diet energy mass. The higher lipid digestibility of insect meals suggests that they provide an energy-rich diet for livestock. The LMM and IMM groups had greater TDN values (61.4% and 61.1%, respectively) than the control group (56.1%). This improvement suggests LMM and IMM diets enhance nutrient absorption. The recent findings indicate Ayaz et al. (2023) that mealworm frass, including insect larvae, dead insects, and their byproducts, improves sheep growth and health. Furthermore, Carrasco and Drewery (2024) reported that beef cattle receiving *Tenebrio molitor* supplements exhibited significantly higher forage OM intake compared to control. This supports the notion that insect protein inclusion enhances not only nutrient digestibility but also voluntary intake, aligning with the improvements observed in DM, OM, and Car digestibility in the LMM and IMM groups. This aligns with our findings, which also showed that mealworm larvae improve nutrient utilization, indicating that insect-based meals enhance feed efficiency and absorption in ruminants.

### Growth performance and feed utilization efficiency

Table 3 shows that lambs that consumed the LMM and IMM diets had higher numerical *P* final body weight, total weight increase, and average daily gain (ADG) than the control group. Moreover, the lack of substantial differences between treatments in concentrate feed mixture (CFM), roughage intake, total dry matter (DM), or total digestible nutrients (TDN) consumption suggests that the incorporation of insect meal did not adversely affect feed palatability or voluntary intake. This finding is consistent with the study by Robles-Jimenez et al. (2025a), which demonstrated that substituting traditional protein sources with yellow mealworm in feedlot lamb diets did not affect feed consumption while preserving growth performance and carcass characteristics. Ayaz et al. (2023) carried out a similar study showing that adding mealworm frass to sheep diets did not reduce feed intake but did improve growth performance and blood parameters, implying more efficient nutrient utilization rather than increased intake. Carrasco and Drewery (2024) reported that in cattle feeding system supplemented with *Tenebrio molitor*, forage organic matter intake and total digestible organic matter intake significantly increased compared to the control, without negatively affecting ruminal fermentation. This suggests that insect protein inclusion can stimulate voluntary intake in ruminants broadly, further supporting the positive growth trends observed in the present study. The IMM group had a higher DCP intake than the other groups, even though the DM and TDN intake were similar across treatments. This could explain why the IMM group grew faster. Increased DCP availability signifies enhanced nitrogen use efficiency and a higher supply of metabolizable protein for tissue accretion. Robles-Jimenez et al. (2025b) corroborated this interpretation by demonstrating through in vitro and in vivo studies that the incorporation of yellow mealworms improved ruminal nutrient digestion and protein utilization in fattening lambs. Better nitrogen balance and protein availability may lead to more muscle growth, even if the amount of food eaten stays the same. The observed positive growth trend may be attributed to the functional components of insect meal. Mealworms provide highly digestible protein and essential amino acids(Inje et al. 2018), alongside bioactive compounds such as chitin. Although chitin is structurally indigestible, moderate levels may modulate ruminal microbiota and improve microbial protein synthesis efficiency. Supporting this concept, Hassanien et al. (2025) reported that dietary inclusion of *Tenebrio molitor* frass improved nutrient digestibility and ruminal fermentation characteristics in goats, resulting in enhanced metabolic efficiency. Likewise, Gonzalez-Ronquillo et al. (2025), through in vitro assessment, demonstrated that dietary mealworm inclusion can positively influence ruminal fermentation patterns, supporting improved nutrient conversion. In addition, the findings of Fukuda et al. (2022), who reported improved body weight responses in beef cattle fed insect-based protein meals, corroborate the present numerical improvements in growth performance. Collectively, these studies indicate that insect-derived protein sources may enhance growth not necessarily by increasing intake, but by improving nutrient digestibility, nitrogen utilization, and metabolic efficiency. Therefore, although the growth improvements in were not statistically significant, the elevated DCP intake in the IMM group, together with evidence from recent literature, supports the hypothesis that insect meal inclusion can enhance protein availability and promote favorable growth responses in lambs. Further studies with larger sample sizes or longer feeding periods may help clarify whether these positive biological trends can achieve statistical significance under practical production conditions.

### Ruminal fermentation

The data presented in Fig. 1 indicate that no significant differences (*P* > 0.05) were detected among treatments in ruminal pH, total volatile fatty acids (TVFA), or ammonia nitrogen (NH₃-N) concentrations at either 0- or 4-hours post-feeding. Maintaining ruminal pH within a physiological range is essential for optimal microbial activity and fiber digestion; therefore, the absence of drastic pH fluctuations confirms that insect meal inclusion did not induce subacute ruminal acidosis or fermentation imbalance. The observed decline in ruminal pH four hours after feeding, accompanied by a concomitant increase in TVFA concentration, reflects the expected postprandial fermentation pattern. This response aligns with the well-established negative correlation between ruminal pH and VFA concentration described by Dijkstra et al. (2012), whereby enhanced microbial fermentation of carbohydrates leads to greater VFA production and a transient reduction in ruminal pH. Importantly, the magnitude of pH decline remained moderate across treatments, indicating that insect meal inclusion did not exacerbate acid accumulation beyond physiological limits. Regarding ammonia nitrogen, the slightly lower NH₃-N concentration observed in the LMM group may suggest improved microbial assimilation of ammonia for microbial protein synthesis rather than excessive protein degradation; however, microbial protein synthesis and rumen microbiota were not directly measured in the present study. Therefore, this interpretation should be considered tentative and requires further confirmation through direct assessment of rumen microbial populations and microbial protein synthesis. Efficient ammonia capture by ruminal microbes is desirable, as it enhances nitrogen utilization efficiency and reduces nitrogen losses. Castillo-Lopez and Domínguez-Ordóñez (2019) emphasized the importance of ammonia-nitrogen in microbial protein synthesis, further supporting the idea that improved ammonia incorporation into microbial protein may explain the moderated NH₃-N concentration observed in the present study. This interpretation is consistent with Jayanegara et al. (2017a), who reported that shifts in proteolytic and nitrogen-degrading microbial populations, including protozoa and proteolytic bacteria, can significantly influence ruminal ammonia dynamics and nitrogen utilization efficiency. Enhanced incorporation of ammonia into microbial protein may therefore explain the moderated NH₃-N concentration observed in the present study. This interpretation is further supported by Robles-Jimenez et al. (2025b), who demonstrated through in vitro and in vivo evaluations that inclusion of *Tenebrio molitor* improved ruminal nutrient digestion without negatively affecting ammonia dynamics. Similarly, Hassanien et al. (2025) reported that dietary inclusion of mealworm frass modulated ruminal fermentation patterns in goats, improving nutrient digestibility while maintaining stable rumen parameters, including NH₃-N concentration.

The stability of TVFA production among treatments suggests that insect meal did not impair carbohydrate fermentation pathways. In fact, insect-derived proteins may support balanced ruminal microbial ecosystems. Gonzalez-Ronquillo et al. (2025) showed in vitro that mealworm inclusion maintained favorable fermentation profiles and did not suppress total VFA production when incorporated into sheep diets. Likewise, Ayaz et al. (2023) observed that mealworm frass inclusion in sheep diets improved growth performance without detrimental effects on metabolic or health indicators, indirectly supporting efficient nutrient fermentation and utilization. Moreover, Robles-Jimenez et al. (2025a) reported that replacing conventional protein sources with yellow mealworm in lamb diets did not adversely affect rumen functionality, growth, or metabolic indicators, indicating that mealworm ingredients can be incorporated without disturbing ruminal homeostasis.

### Blood indicators

Table 4 presents the effects of larval meal (LMM) and insect meal (IMM) on serum biochemical parameters. Overall, no substantial alterations were observed in most evaluated blood markers across experimental groups, indicating that insect inclusion in ruminant diets did not disrupt systemic metabolic balance or hepatic function, supporting its physiological safety. Comparable outcomes were reported by Robles-Jimenez et al. (2025a), who found that replacing conventional protein sources with yellow mealworm in lamb diets did not adversely affect blood parameters or metabolic health. Likewise, their complementary in vitro and in vivo investigation (Robles-Jimenez et al. 2025b) further confirmed that *Tenebrio molitor* could be incorporated into lamb diets without detrimental effects on blood metabolites.

Recent findings by Carrasco and Drewery (2024) further support these observations, shows that Mealworm supplementation in beef cattle did not significantly alter serum biochemical parameters, including albumin, globulin, and glucose concentrations. This finding aligns with the current study’s conclusion that insect meal inclusion does not disrupt systemic metabolic homeostasis.

Serum albumin concentration was higher in the control group than in the LMM and IMM groups. Albumin plays a critical role in maintaining osmotic pressure and serves as an indicator of nutritional status and hepatic function. Despite the numerically reduced in insect-fed groups, albumin values remained within physiological ranges, suggesting preserved hepatic functional. Similar observations were noted by Ayaz et al. (2023), who reported that inclusion of mealworm frass in sheep diets did not negatively affect serum biochemical indices. Furthermore, Hassanien et al. (2025) observed stable blood metabolite profiles in goats receiving *Tenebrio molitor* frass, confirming that insect-derived ingredients do not compromise liver-related biochemical parameters.

Conversely, globulin concentrations were significantly (*P* = 0.012) elevated in both LMM and IMM groups compared to the control group. Increased globulin levels are commonly associated with enhanced synthesis of immune-related proteins and may reflect activation or modulation of humoral immune responses. This effect may be linked to bioactive constituents present in insect meals, particularly chitin. Previous investigations (Hahn et al. 2020; Van Huis and Gasco 2023; Abenaim and Conti 2025) suggested that chitin and related bioactive compounds in insect-based diets can stimulate immune function and enhance host defense mechanisms. Supporting this interpretation, Ayaz et al. (2023) documented alterations in hematological and serum parameters indicative of immune modulation without pathological consequences. Carrasco and Drewery (2024) also reported that serum globulin concentrations increased in cattle supplemented with insect protein, supporting the hypothesis that the elevated globulin levels observed in the current study are likely due to adaptive immunological modulation, without adverse effects. This support suggests that insect-derived ingredients, such as *Tenebrio molitor*, may beneficially stimulate immune responses in ruminants.

A significant (*P* < 0.0001) reduction in serum glucose concentration was observed in the IMM group compared with the control and LMM groups. This decline may indicate enhanced peripheral glucose utilization or improved metabolic efficiency rather than impaired energy balance, particularly in the absence of adverse health indicators. The potential role of chitin in glucose metabolism provides a plausible mechanistic explanation. Son et al. (2021) reported that chitin may support digestion and facilitate glucose absorption, thereby influencing systemic glucose dynamics. Similarly, Radwan et al. (2023) found that inclusion of mealworm frass in rabbit diets resulted in reduced blood glucose concentrations, suggesting a regulatory effect of insect-derived components on carbohydrate metabolism. Carrasco and Drewery (2024) also highlighted that insect meal inclusion in cattle diets resulted in lower serum glucose levels, which mirrors the findings of the present study. This supports the interpretation that insect-based proteins can modulate glucose metabolism, possibly by improving energy utilization and promoting a more efficient metabolic profile. Although the exact mechanisms remain to be clarified, the compatibility of *Tenebrio molitor* with normal metabolic processes is further supported by Gonzalez-Ronquillo et al. (2025), who demonstrated in vitro that dietary mealworm inclusion maintained favorable fermentation characteristics in sheep diets, indicating that insect proteins integrate effectively within ruminant digestive and metabolic pathways. A closer comparative evaluation with previously published studies further strengthens the interpretation of the present findings. In the study of Ayaz et al. (2023), dietary inclusion of mealworm frass in sheep did not result in detrimental alterations in serum total protein, albumin, or glucose concentrations. Although mild modulation of hematological and biochemical indices was observed, suggesting adaptive physiological responses rather than metabolic disturbance. Similarly, Robles-Jimenez et al. (2025a) reported stable albumin, globulin, and glucose concentrations following mealworm inclusion in lamb diets, indicating preserved hepatic and metabolic function. Findings from complementary investigation (Robles-Jimenez et al. 2025b) further confirmed metabolic stability alongside maintained growth performance. The elevation in globulin observed in the present study is consistent with the findings of Hassanien et al. (2025), who reported modifications in blood metabolites without evidence of inflammatory stress, suggesting controlled immunological modulation. Moreover, the reduction in glucose concentration parallels the observations of Radwan et al. (2023), indicating a consistent trend toward moderated glycemic levels following insect-derived ingredient inclusion. Although species and dietary contexts differ, the convergence of evidence across studies supports the hypothesis that insect-based proteins can modulate immune and metabolic responses without compromising systemic health. Taken together, the biochemical responses in Table 4 demonstrate that with the inclusion of insect in ruminant diets preserves systemic metabolic homeostasis while potentially exerting functional effects on immune activity and glucose regulation. The consistency of these findings with previous reports (Hahn et al. 2020; Son et al. 2021; Radwan et al. 2023; Van Huis and Gasco 2023; Ayaz et al. 2023; Abenaim and Conti 2025; Robles-Jimenez et al. 2025a, b; Gonzalez-Ronquillo et al. 2025; Hassanien et al. 2025) strengthens the evidence supporting the safe and functional incorporation of *Tenebrio molitor*–based ingredients in ruminant nutrition.

### Economic efficiency

The economic evaluation presented in Table 5 indicates that both LMM and IMM diets generated higher numerical returns on investment and improved profitability compared with the control group, although differences were not statistically significant. Despite the lack of statistical significance, the consistent numerical improvement across economic indicators suggests practical relevance, particularly in tropical production systems where feed efficiency strongly influences profitability. Uniform lamb acquisition and maintenance costs across treatments confirm that the observed economic variation was primarily driven by dietary effects. The improved economic trend, especially in the IMM group, may be attributed to enhanced feed conversion efficiency or superior nutrient utilization. Improved digestibility and metabolic efficiency reduce feed cost per unit of gain, thereby enhancing economic return. These findings align with Hassanien et al. (2025), who reported improved nutrient digestibility and productive responses in goats receiving *Tenebrio molitor* frass. Similarly, Ayaz et al. (2023) observed improved growth performance in sheep without negative biochemical consequences, supporting the notion that insect-derived ingredients can sustain productivity without increasing health-related costs. In addition, Robles-Jimenez et al. (2025a, b) demonstrated maintained growth performance, carcass traits, and metabolic stability in lambs fed yellow mealworm, reinforcing the economic feasibility of insect-based protein inclusion. The favorable ruminal fermentation patterns reported by Gonzalez-Ronquillo et al. (2025) provide additional biological support for improved feed efficiency and economic performance. Importantly, the economic outcomes appear biologically coherent with the metabolic adaptations observed in Table 4. Such metabolic stability, combined with improved nutrient utilization, provides a mechanistic explanation for the positive economic trend observed in LMM and IMM groups. Improved metabolic efficiency typically enhances feed conversion ratio, thereby reducing feed cost per unit of weight gain. Consequently, the improved profitability indicators observed numerically in the insect-fed groups may represent a direct outcome of enhanced biological efficiency rather than incidental variation. These findings are further supported by Carrasco and Drewery (2024), although a formal economic analysis was not conducted, the observed improvements in intake and digestibility. These findings suggest that insect protein supplementation, such as from *Tenebrio molitor* and black soldier fly larvae, could enhance nutrient utilization efficiency, potentially reducing feed costs per unit of gain. The alignment between physiological stability, metabolic adaptation, and economic performance strengthens the interpretation that insect-derived protein sources can simultaneously preserve animal health and support production efficiency. Under tropical production systems, where feed resources may be limited and production margins are highly sensitive to feed efficiency, insect-derived protein ingredients may offer strategic advantages. The ability of *Tenebrio molitor*–based meals to maintain metabolic stability, support digestive efficiency, and sustain productive performance suggests their potential contribution to economic resilience and sustainability. Additionally, insect production systems generally require fewer environmental resources than conventional ingredients, further enhancing their long-term viability.

## Conclusions

Dietary supplementation with Tenebrio molitor larvae meal or adult insect meal improved selected nutrient digestibility coefficients, particularly DM, OM, EE, carbohydrate digestibility, and TDN, without adverse effects on most blood biochemical parameters. Growth performance and economic return showed only numerical improvements and did not differ significantly among treatments. Therefore, Tenebrio molitor may represent a promising sustainable feed supplement for growing lambs; however, further studies with larger sample size, replicated pens, higher inclusion levels, and direct rumen microbial measurements are required before firm practical recommendations can be made.

## Data Availability

Not applicable.

## Declarations

### Declarations generative AI

Throughout the manuscript preparation, authors conducted Chat GPT to improve English grammar in some parts. After using it, the authors reviewed it prior to submission.
